# T2 mapping for quantitative assessment of ankle cartilage of weightlifters

**DOI:** 10.1038/s41598-023-46259-w

**Published:** 2023-11-06

**Authors:** Weibiao Wu, Zhuanzhuan Kang, Di Mu, Huiyu Zhao, Feng Yang

**Affiliations:** Radiology Department, Central Hospital Affiliated to Shenyang Medical College, No.5 NanQiXi Road, TieXi District, Shenyang, Liaoning 110024 People’s Republic of China

**Keywords:** Health care, Medical research

## Abstract

The research into the prevention of sports injuries among the population, particularly juveniles, has become crucial due to the increasing participation in physical exercises like fitness. To assess the difference in T2 values of ankle talar cartilage between weightlifters and healthy volunteers using quantitative magnetic resonance imaging (MRI) technique T2 mapping. Study design: Prospective. Prospective evaluation of T2 values of ankle cartilage of 50 weightlifters (30 adults and 20 juveniles) and 100 healthy volunteers (80 adults and 20 juveniles) using Siemens 3.0 T MRI with PDWI, T1WI, and T2 mapping sequences. Three physicians manually divided the talar cartilage of the ankle joint into six regions of interest. Three physicians utilized the anterior and posterior cut edges of the tibial cartilage as markers to identify the corresponding anterior and posterior cut edges of the talar cartilage on the sagittal MRI images. The medial and lateral sides were defined as half of the talar articular surface on the coronal plane. Differences in T2 values in each cartilage region were compared using independent sample T test or Mann–Whitney *U* test. The T2 values of talar cartilage were significantly increased in the athlete group relative to the volunteer group (35.11 and 31.99, P < 0.001), with the most significant difference observed in the juvenile athlete group compared to the volunteer group (34.42 and 28.73, P < 0.001). There was a significant difference in the T2 value of ankle talar cartilage between weightlifters and healthy volunteers, and juveniles may be more vulnerable to overuse sports injuries. This study contributes to understanding the cartilage health of juvenile athletes and the prevention of sports injuries.

## Introduction

Active participation in sports offers numerous benefits, and promoting sports involvement is of paramount importance to modern society^1^. Sports participation has been found to have a positive impact on cardiovascular health, self-efficacy, self-perceived quality of life^2,3^, and employment opportunities^4^. However, despite the many benefits of physical activity, there are also inherent risks of injury associated with sports participation^5^. Sustaining a sports injury can negatively impact an individual's motivation to continue participating in sports and can also affect team and individual sports performance^6^. Therefore, preventive measures are crucial to mitigating the harmful effects of sports injuries on individuals and society^7^. With an increasing number of youths engaging in sports and recreational activities, there has been a rise in both acute and overuse injuries^8^. Sports injuries account for the largest proportion of all injuries in adolescents and have a significant impact on adults^9^. There are physical and physiological differences between adolescents and adults that may make children more susceptible to injuries^8^. More than 50% of sports injuries occur in the lower extremities, with ankle sprains accounting for approximately 25% of all sports injuries. Additionally, post-traumatic ankle osteoarthritis progresses more rapidly in younger age groups^10^. In recent years, an increasing number of athletes have been training at a much younger age with high intensity or participating in multiple sports within a season, leading to an elevated chance of suffering an acute injury while increasing the risk of overuse injuries. Across all age groups, emergency room visits for sports-related injuries peak at ages 5–14 years and decrease with age. School-age children (5–12 years), adolescents (13–18 years), and young adults (18–24 years) have the highest rates of sports injury visits^11^. In weightlifters, there is an increase in cartilage thickness in the weight-bearing and non-weight-bearing zones of the knee, and these changes are negatively correlated with the time of training initiation. Athletes who encounter high-impact loads in childhood, adolescence, and adulthood may suffer from cartilage damage, and there is growing evidence that chondrocyte death is critical in the early stages of osteoarthritis^10^. Therefore, it is crucial to choose a diagnostic method that allows for rapid, accurate, highly sensitive, and noninvasive early detection of cartilage lesions in the joint^12^. Numerous reports have demonstrated that component MRI assessment techniques can detect early degenerative changes in articular cartilage before morphological manifestations are detected by conventional MRI. T2 mapping, a promising component MRI technique, can assess articular cartilage in osteoarthritis^13–15^. Increased cartilage T2 relaxation time is associated with increased water content, type II collagen deficiency, and anisotropy of collagen fibers^16–19^. T2 mapping can be used to assess and monitor the properties of the repaired tissue based on the interaction between water molecules and collagen networks in regenerating cartilage^20–23^. Good collagen tissue is considered mature repair tissue with good clinical relevance^24^, while poor cartilage tissue suggests poor clinical outcomes^25,26^. Therefore, early prediction and diagnosis of cartilage may prevent or delay the onset of osteoarthritis^27^.

With the incidence rate of sports injuries on the rise, particularly among juveniles, there is an urgent need to detect changes that occur before the onset of sports injuries to prevent them. This paper aims to explore the category of sports injuries known as 'overuse injury' and its impact on both adults and juveniles using T2 mapping technology. A thorough understanding of the study methodology can assist clinicians in evaluating whether a study finding is valid and applicable to the patient's context.

## Materials and methods

### Study population

Informed consent was obtained from all participants after fully explaining the nature of the study. This study was approved by the Shenyang Medical College Institutional Review Board. Volunteers were recruited in non-clinical settings through advertising, the Internet, public interest, and by word-of-mouth.

The study population consisted of 50 weightlifters from sports centers who were recruited from October 2021 to January 2022 (athlete group) and 100 healthy volunteers who were recruited from October 2021 to May 2022 (long-term sedentary, volunteer group). The inclusion criteria for the study were age between 15 and 30 years, a BMI range of 18.5 to 27.9 kg/m^2^, no current ankle or other joint swellings, pain, or stiffness, and no previous history of ankle fracture, deformity, or surgery. Exclusion criteria included poor quality MRI images (artifacts, poor display, etc.) and abnormal lesion signal on routine MRI findings.

**Figure 1:**
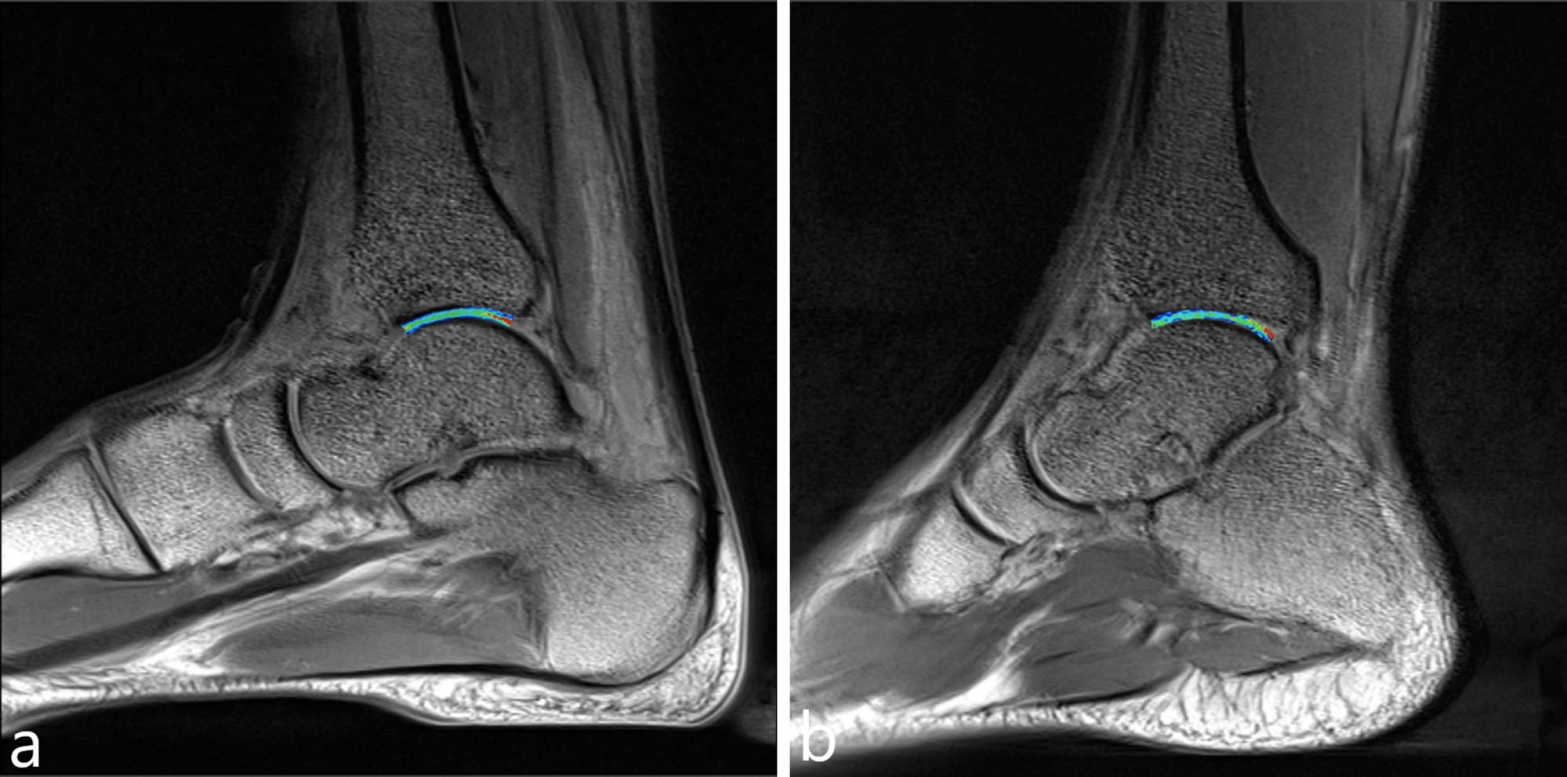
(**a**) is the T2 mapping of the ankle joint of a 25-year-old male volunteer, (**b**) is the T2 mapping of the ankle joint of a 25-year-old female volunteer.

**Figure 2:**
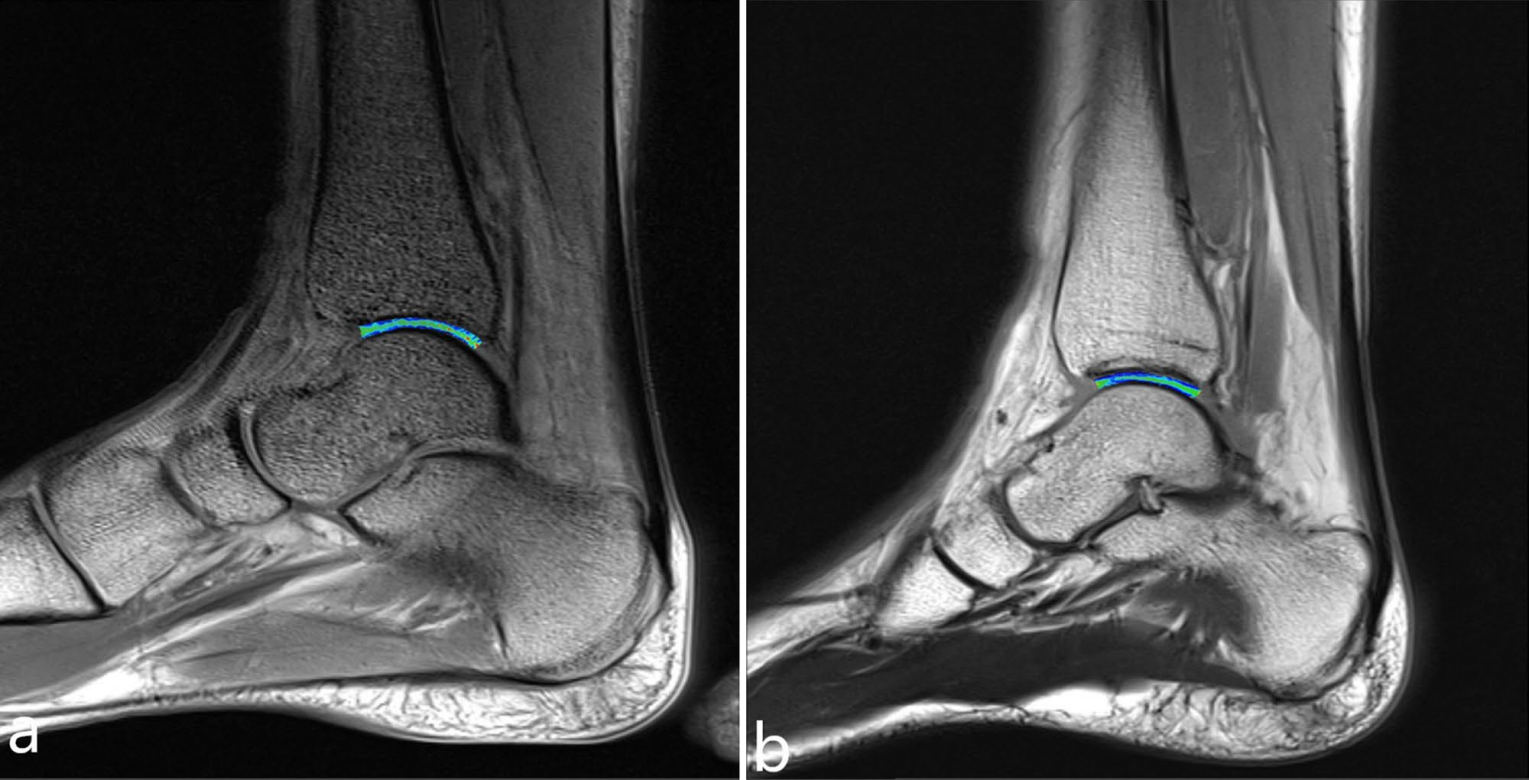
(**a**) is the T2 mapping of the ankle joint of a 17-year-old male volunteer, (**b**) is the T2 mapping of the ankle joint of a 17-year-old female volunteer.

A total of 100 volunteers participated in this study, with a mean age of 23.3 ± 4.0 years and an age range of 15–30 years. Among these volunteers, 80 were adults (40 males and 40 females) with a mean age of 25.1 ± 1.9 years and an age range of 18–30 years (Fig. 1a,b). The remaining 20 participants were juveniles (10 males and 10 females) with a mean age of 16.2 ± 0.9 years and an age range of 15–17 years (Fig. 2a,b). The mean BMI of the volunteers was 20.7 ± 1.7 kg/m^2^, with a range of 18.5–27.9 kg/m^2^. The mean BMI for adult volunteers was 20.9 ± 1.8 kg/m^2^ and for juvenile volunteers, it was 20.0 ± 0.7 kg/m^2^.

**Figure 3:**
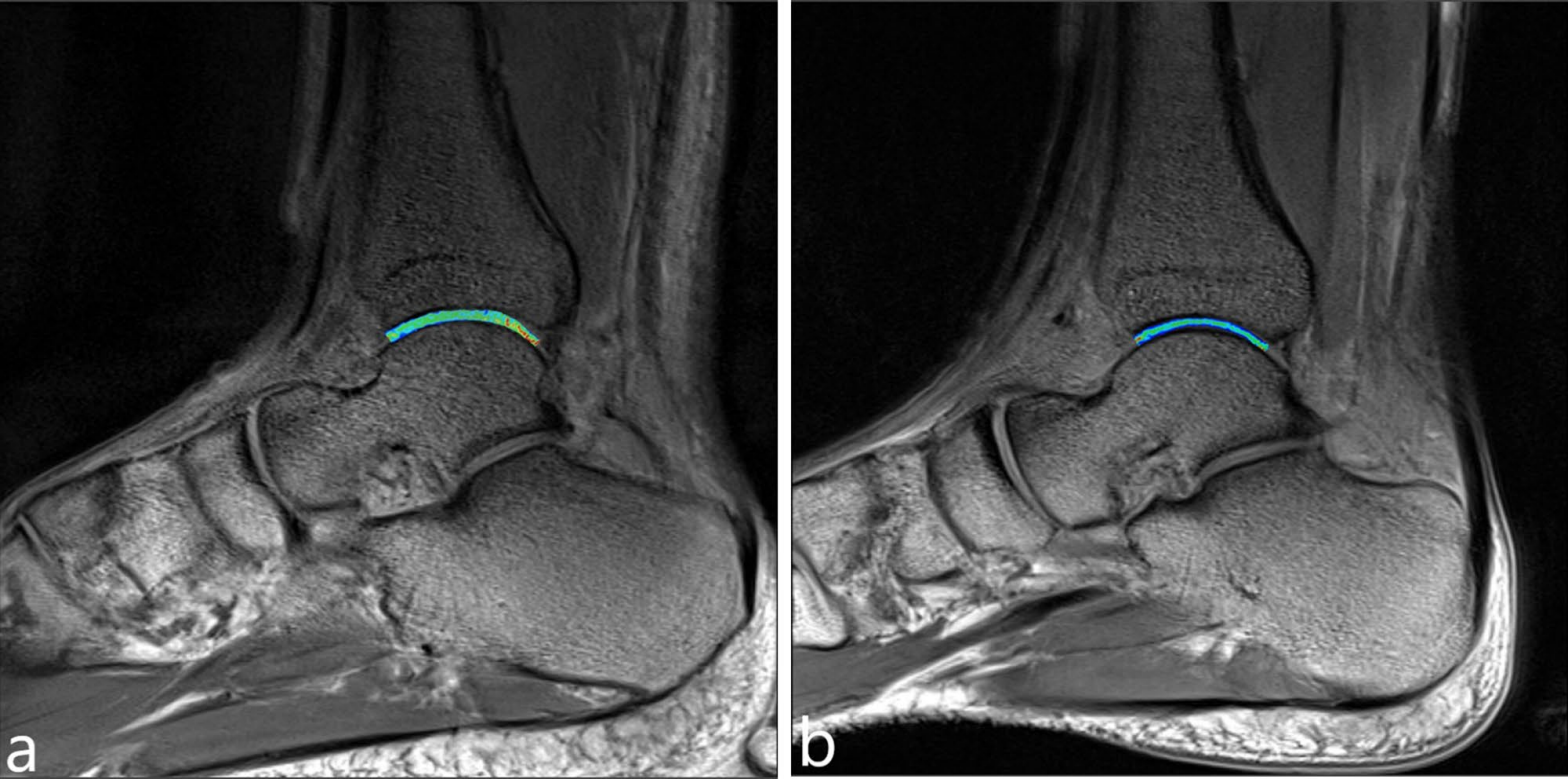
(**a**) is the T2 mapping of the ankle joint of a 25-year-old male athlete, (**b**) is the T2 mapping of the ankle joint of a 25-year-old female athlete.

**Figure 4:**
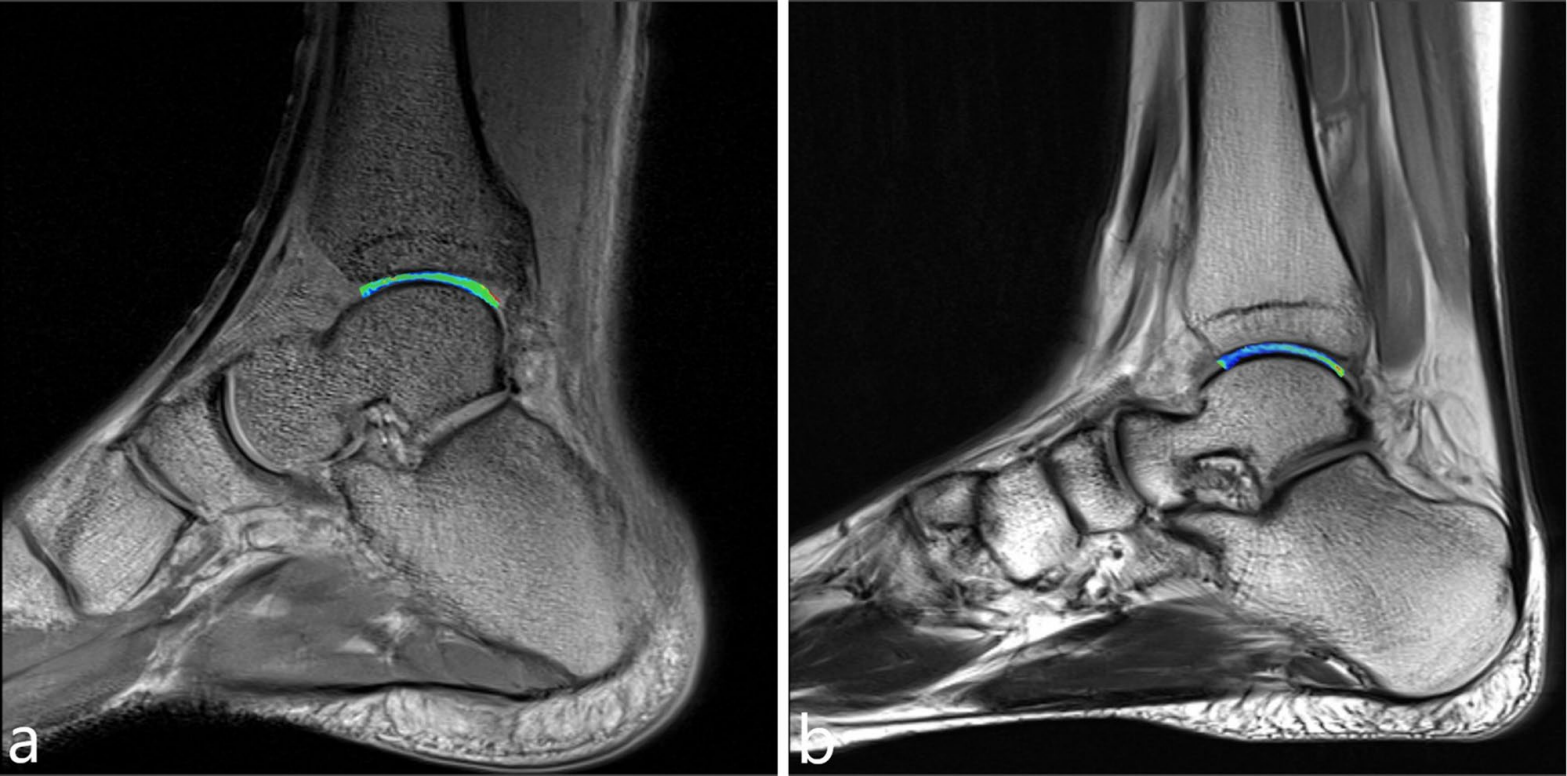
(**a**) is the T2 mapping of the ankle joint of a 17-year-old male athlete, (**b**) is the T2 mapping of the ankle joint of a 17-year-old female athlete.

A total of 50 athletes took part in the study, with a mean age of 19.5 ± 3.7 years and an age range of 15–30 years. Among these athletes, 30 were adults (15 males and 15 females) with a mean age of 21.6 ± 3.5 years and an age range of 18–30 years (Fig. 3a,b), while the remaining 20 were juveniles (10 males and 10 females) with a mean age of 16.6 ± 0.5 years and an age range of 15–17 years (Fig. 4a,b). The mean BMI of the athletes was 22.6 ± 2.0 kg/m^2^, ranging from 18.5 to 27.9 kg/m^2^. The BMI for adult athletes was 23.1 ± 2.1 kg/m^2^, while for juvenile athletes, it was 21.9 ± 1.9 kg/m^2^. Athletes followed a comprehensive year-round training program and trained approximately 18 times per week for 2 h each session. The average length of training for athletes was 5.3 ± 2.1 years, with adult athletes having an average length of training of 6.5 ± 1.3 years and juvenile athletes having an average length of 3.6 ± 1.9 years.

### Image acquisition

**Figure 5:**
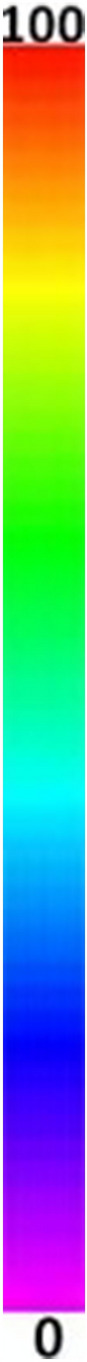
Color card of T2 mapping.

To minimize the impact of daily activities on the T2 values of ankle cartilage, all MRI scans were conducted in the morning. Two weeks before the start of the study, all volunteers were instructed to limit their weight-bearing activities and stand or walk for an hour before the study but not sit or lie down. Scans were performed using a 3.0 T-MRI scanner (Siemens Verio 3.0 T, Germany) fitted with an 8-channel dedicated ankle imaging coil. Participants underwent ankle MRI imaging while lying supine with their foot at a 90-degree angle to the long axis of the lower leg. The imaging protocol included sagittal fat-suppressed proton density-weighted spin-echo sequence, coronal fat-suppressed proton density-weighted spin-echo sequence, axial fat-suppressed proton density-weighted spin-echo sequence, sagittal T1-weighted spin-echo sequence, and sagittal T2 mapping sequence. Details of the MRI protocol are presented in Table 1. T2 pseudo-color images were obtained using pixel-level single-exponential non-negative least squares fitting analysis. Color card of T2 mapping (Fig. 5).

**Table 1:** MRI sequence parameters for each imaging sequence.

Sequence	T1-sag	pd-fs-sag	pd-fs-cor	pd-fs-tra	T2 map
TR (ms)	810	3000	3000	2700	1540
TE (ms)	14	40	37	40	13.8
FOV (mm)	160	160	160	140	160
Distance factor (%)	10	10	10	10	20
Slices	20	20	20	20	20
Slice thickness (mm)	3	3	3	3	3
Phase oversampling (%)	80	80	80	50	50
Flip angle (deg)	150	150	150	150	180
Imaging time (min:s)	2:02	1:59	1:59	2:44	6:27

### Data analysis

**Figure 6:**
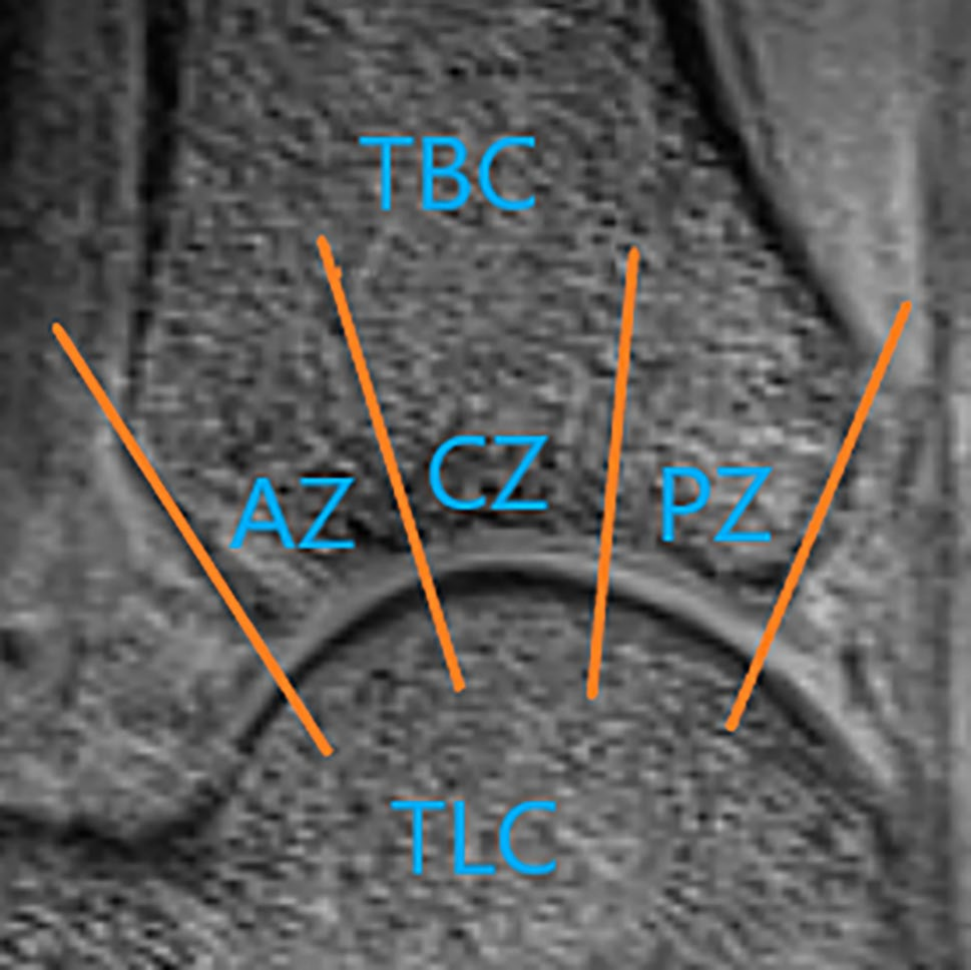
In the sagittal plane, the TBC and TLC was divided into three zones: AZ, CZ, PZ.

**Figure 7:**
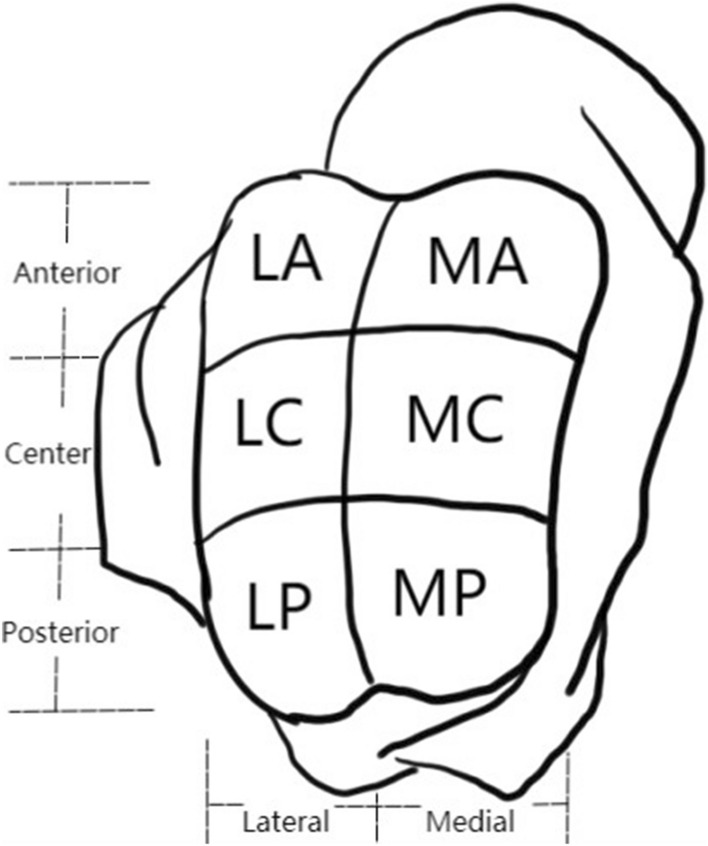
In the each sagittal plane, TLC was divided into six regions: MA, MC, MP, LA, LC, LP.

Three radiologists (names redacted), each with 3 years of experience in musculoskeletal MRI and 3 months of training in cartilage segmentation, retrospectively and independently analyzed the MRI images. During the analysis, they were blinded to the patient's information. The observers used the anterior and posterior cut edges of the tibial cartilage (TBC) as markers for the anterior and posterior cut edges of the talar cartilage (TLC) on the sagittal MRI images. The tibial-talar joint was divided into three equal regions: anterior zone (AZ), central zone (CZ), and posterior zone (PZ), as illustrated in Fig. 6. Virtual lines were drawn between the three zones using the tool and saved on a Siemens Syngo workstation. The cartilage was segmented independently by the three observers. The medial and lateral sides of the talar articular surface on the coronal plane were included, encompassing 3–4 effectively contiguous sagittal planes. The optimal level was determined based on the effective contiguous layers of the T2 map, with one optimal level obtained on each of the medial and lateral sides. As such, six regions of interest (ROIs) were obtained for each TLC: medial anterior (MA), medial center (MC), medial posterior (MP), lateral anterior (LA), lateral center (LC), and lateral posterior (LP), as depicted in Fig. 7. Three observers independently obtained T2 relaxation times of the ankle TLC and the six ROIs from the processed sagittal plane T2 maps. The three T2 values obtained by each observer for each region were averaged to compare cartilage T2 values between regions. The average T2 value of the six ROIs was used to determine T2 values for TLC. Oblique articular surfaces, such as the medial and lateral ankle surfaces of the tibia, talar, and fibula, were excluded from the analysis to avoid any partial volume averaging effects, and synovial fluid or subchondral bone were not included in the ROIs. When it was challenging to determine the division between TBC and TLC, each TBC and TLC was manually divided into two equal-width cross-sections. The rate of increase in the T2 value of each cartilage area in the athlete group relative to the T2 value of the corresponding cartilage area in the healthy volunteer group was calculated using the formula: growth rate = (T2 value of each cartilage area in the athlete group − T2 value of the corresponding cartilage area in the volunteer group) × 100%/T2 value of the corresponding cartilage area in the volunteer group.

### Statistical analysis

Our research employed random sampling as the selected sampling method to ensure equitable and unbiased representation of the population. Nevertheless, we acknowledge that we did not explicitly perform a sample calculation to determine the sample size due to practical constraints, including resource limitations, time constraints, and limited access to the entire population.

All statistical analyses were conducted using SPSS version 26.0 software. Standard demographic data, such as age and BMI, were reported as mean ± standard deviation for both groups. Interobserver and intraobserver agreements were evaluated using ICCs. A significance level of P < 0.05 was considered statistically significant.

The Shapiro–Wilk test was applied to determine if the data from the athlete and volunteer groups were normally distributed. If the data adhered to a normal distribution, it was expressed as mean ± standard deviation. Otherwise, median (the first quartile, the third quartile) was used to represent the data. Furthermore, independent-sample T-tests or Mann–Whitney U tests were used to verify whether there were any significant differences in TLC and ROI between the two groups.

To establish intra-observer reproducibility, the ICC was employed to assess inter-observer reliability for three observers, **, **, and **. To evaluate reliability between **, **, and **, thirty images from the athlete group were randomly selected to measure T2 values. Three weeks later, observers **, **, and ** were re-measured for the same 30 athlete group images to assess the reliability of observers **, **, and **.

### Ethics approval and consent to participate

All methods were carried out in accordance with relevant guidelines and regulations. All experimental protocols were approved by Shenyang Medical College Institutional Review Board. All subjects and/or their legal guardian(s) consent to participate this study. This study was approved by the Shenyang Medical College Institutional Review Board and all participants consent to participate.

### Informed consent

All included participants and/or their legal guardian(s) gave their oral and written informed consent.

## Results

The T2 values for each ROI and the talar cartilage were evaluated for normality in the group of 100 volunteers, and three ROIs were observed to conform to a normal distribution (P > 0.05): medial anterior talar (P = 0.350), lateral anterior talar (P = 0.216), and lateral center talar (P = 0.666). Three ROIs and the talar cartilage did not conform to a normal distribution (P < 0.05): medial center talar (P = 0.040), medial posterior talar (P < 0.001), lateral posterior talar (P < 0.001), and talar cartilage (P = 0.038). In the group of 50 athletes, the T2 values for each ROI and talar cartilage were also tested for normality, and six ROIs and the talar cartilage conformed to a normal distribution (P > 0.05): medial anterior talar (P = 0.416), medial center talar (P = 0.165), medial posterior talar (P = 0.273), lateral anterior talar (P = 0.053), lateral center talar (P = 0.244), lateral posterior talar (P = 0.969), and talar cartilage (P = 0.172). Detailed data are presented in Table 2.

**Table 2:** P-value of normality test for each region for athletes and volunteers.

Variables	Athlete P-value	Variables	Volunteer P-value
MA	0.416	MA	0.350
MC	0.165	MC	0.040
MP	0.273	MP	< 0.001
LA	0.053	LA	0.216
LC	0.244	LC	0.666
LP	0.969	LP	< 0.001
TLC	0.172	TLC	0.038

When comparing the ROI and T2 values between the athlete group and the volunteer group, significant differences (P < 0.05) were observed in the T2 values of all six ROIs and the cartilage of the talar in the athlete group compared to the volunteer group. The ROIs included the medial anterior talar (34.22/32.06, P = 0.001), medial center talar (34.36/30.11, P < 0.001), medial posterior talar (36.40/32.61, P < 0.001), lateral anterior talar (33.77/31.52, P < 0.001), lateral center talar (34.89/30.62, P < 0.001), and lateral posterior talar (37.03/32.16, P < 0.001), as well as the talar cartilage (35.11/31.99, P < 0.001). Detailed data are presented in Table 3.

**Table 3:** Comparison of T2 values of ankle talar cartilage between the athlete group and the volunteer group.

Variables	T2 value		Difference	Rate (%)	Significance (P value)
	Athlete group	Volunteer group			
MA	34.22 ± 1.49	32.06 ± 2.86	2.16	6.7	0.001^b^
MC	34.36 ± 1.26	30.11 (28.43, 32.27)	4.25	14.1	< 0.001^b^
MP	36.40 ± 1.46	32.61 (29.56, 36.14)	3.79	11.6	< 0.001^b^
LA	33.77 ± 1.91	31.52 ± 2.80	2.25	7.1	< 0.001^b^
LC	34.89 ± 1.25	30.62 ± 3.18	4.27	13.9	< 0.001^b^
LP	37.03 ± 0.79	32.16 (30.48, 35.74)	4.87	15.1	< 0.001^b^
TLC	35.11 ± 0.91	31.99 (29.85, 33.62)	3.12	9.8	< 0.001^b^

P^a^ is independent sample T test, and P^b^ is Mann–Whitney *U* test.

When comparing the ROI and T2 values between adult athletes and adult volunteers, there were significant increases (P < 0.05) in T2 values observed in all six ROIs and talar cartilage of the athlete group compared to the volunteer group. The ROIs included medial anterior talar (35.36/32.53, P = 0.002), medial center talar (35.01/30.43, P < 0.001), medial posterior talar (37.10/34.30, P = 0.001), lateral anterior talar (34.63/32.16, P = 0.005), lateral center talar (35.30/31.43, P < 0.001), and lateral posterior talar (37.45/32.23, P < 0.001), as well as the talar cartilage (35.81/32.41, P < 0.001). Detailed data are presented in Table 4.

**Table 4:** Comparison of ankle talar cartilage T2 values between adult athletes and adult volunteers.

Variables	T2 value		Difference	Rate (%)	Significance (P value)
	Athlete group	Volunteer group			
MA	35.36 ± 1.06	32.53 ± 2.92	2.83	8.7	0.002^b^
MC	35.01 ± 0.79	30.43 (28.95, 32.79)	4.58	15.1	< 0.001^b^
MP	37.10 ± 0.92	34.30 (31.01, 36.63)	2.80	8.2	0.001^b^
LA	34.63 ± 1.47	32.16 ± 2.68	2.47	7.7	0.005^a^
LC	35.30 ± 1.28	31.43 ± 2.98	3.87	12.3	< 0.001^a^
LP	37.45 ± 0.54	32.23 (31.19, 36.73)	5.22	16.2	< 0.001^b^
TLC	35.81 ± 0.42	32.41 ± 1.91	3.40	10.5	< 0.001^b^

P^a^ is independent sample T test, and P^b^ is Mann–Whitney *U* test.

When comparing the ROI and T2 values between immature athletes and immature volunteers, significant increases (P < 0.05) were observed in the T2 values of all six ROIs and talar cartilage of the athlete group compared to the volunteer group. The ROIs were medial anterior talar (33.07/30.19, P < 0.001), medial center talar (33.71/28.01, P < 0.001), medial posterior talar (35.70/28.58, P < 0.001), lateral anterior talar (33.63/29.08, P < 0.001), lateral center talar (34.47/27.37, P < 0.001), and lateral posterior talar (36.62/29.64, P < 0.001), as well as the talar cartilage (34.42/28.73, P < 0.001). Detailed data are presented in Table 5.

**Table 5:** Comparison of T2 values of ankle talar cartilage between Juvenile athletes and Juvenile volunteers.

Variables	T2 value		Difference	Rate (%)	Significance (P value)
	Athlete group	Volunteer group			
MA	33.07 ± 0.80	30.19 ± 1.61	2.88	9.5	< 0.001^a^
MC	33.71 ± 1.34	28.01 (25.68, 29.85)	5.70	20.3	< 0.001^b^
MP	35.70 ± 1.61	28.58 ± 1.32	7.12	24.9	< 0.001^a^
LA	33.63 (32.20, 34.33)	29.08 (27.48, 30.49)	4.55	15.6	< 0.001^b^
LC	34.47 ± 1.14	27.37 ± 1.35	7.10	25.9	< 0.001^a^
LP	36.62 ± 0.81	29.64 ± 1.32	6.98	23.5	< 0.001^a^
TLC	34.42 ± 0.71	28.73 ± 1.02	5.69	19.8	< 0.001^a^

P^a^ is independent sample T test, and P^b^ is Mann–Whitney *U* test.

When comparing the T2 values of each ROI and talar cartilage between adult volunteers and juvenile volunteers, significant increases (P < 0.05) were observed in all six ROIs and talar cartilage of the athlete group compared to the volunteer group. The ROIs were medial anterior talar (32.53/30.19, P = 0.001), medial center talar (30.43/28.01, P < 0.001), medial posterior talar (34.30/28.58, P < 0.001), lateral anterior talar (32.16/29.08, P < 0.001), lateral center talar (31.43/27.37, P < 0.001), and lateral posterior talar (32.23/29.64, P < 0.001), as well as the talar cartilage (32.41/28.73, P < 0.001). Detailed data are presented in Table 6.

**Table 6:** Comparison of T2 values of ankle talar cartilage between adult volunteers and Juvenile volunteers.

Variables	T2 value		Difference	Rate (%)	Significance (P value)
	adult group	Juvenile group			
MA	32.53 ± 2.92	30.19 ± 1.61	2.34	7.8	0.001^b^
MC	30.43 (28.95, 32.79)	28.01 (25.68, 29.85)	2.42	8.6	< 0.001^b^
MP	34.30 (31.01, 36.63)	28.58 ± 1.32	5.72	20.0	< 0.001^b^
LA	32.16 ± 2.68	29.08 (27.48, 30.49)	3.08	10.6	< 0.001^b^
LC	31.43 ± 2.98	27.37 ± 1.35	4.06	14.8	< 0.001^b^
LP	32.23 (31.19, 36.73)	29.64 ± 1.32	2.59	8.7	< 0.001^b^
TLC	32.41 ± 1.91	28.73 ± 1.02	3.68	12.8	< 0.001^b^

P^a^ is independent sample T test, and P^b^ is Mann–Whitney *U* test.

**Figure 8:**
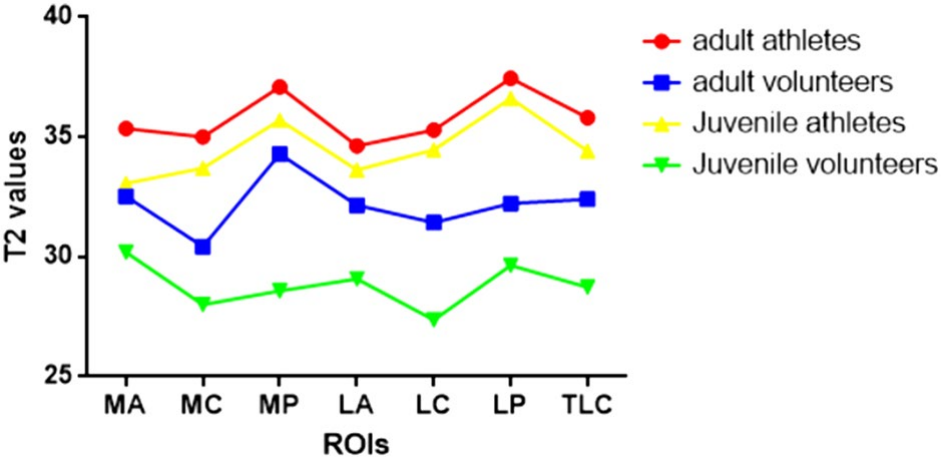
The linear graph is represented by the average (or median, if the normal distribution is not satisfied) of the T2 value of ROI.

**Figure 9:**
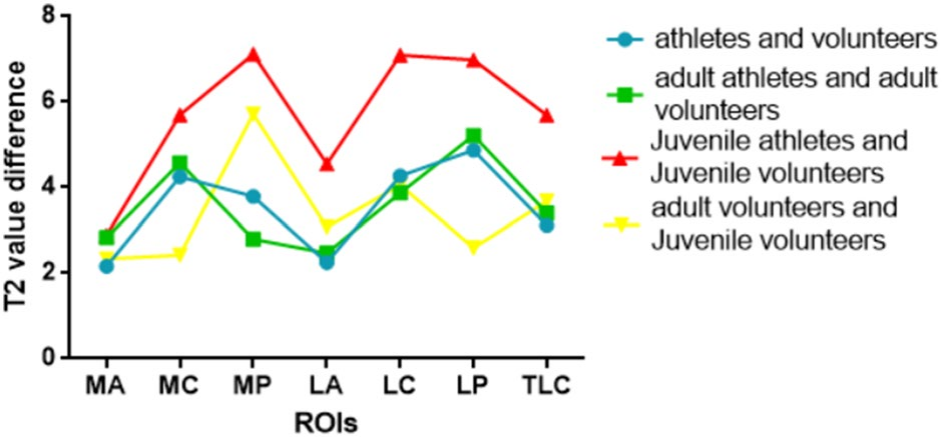
The linear graph is represented by the inter-group difference of the T2 average (or median, if the normal distribution is not satisfied) of ROI.

**Figure 10:**
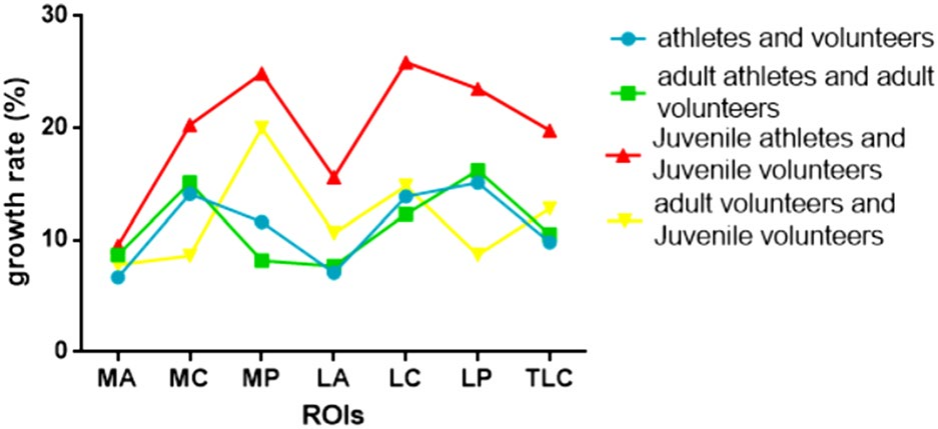
The linear graph is represented by the inter-group growth rate of the T2 average (or median, if the normal distribution is not satisfied) of ROI.

When comparing athletes and volunteers, the largest difference in the talar region was observed in the lateral posterior with the highest growth rate (37.03/32.16, 15.1%). Similarly, in adult athletes and adult volunteers, the largest difference was found in the lateral posterior region of the talar with the highest growth rate (37.45/32.23, 16.2%). However, for juvenile athletes and juvenile volunteers, the largest difference was seen in the medial posterior region of the talar (35.70/28.58), while the highest growth rate occurred in the lateral center region of the talar (25.9%). In the comparison between adult and juvenile volunteers, the largest difference was observed in the medial posterior region of the talar, with the highest growth rate (34.30/28.58, 20.0%). T2 value of ankle cartilage of four different populations (Fig. 8 and Table 7). The difference and growth rate change between the control groups (Figs. 9, 10).

**Table 7:** T2 value of ankle cartilage of four different populations.

Variables	T2 value			
	adult athletes	adult volunteers	Juvenile athletes	Juvenile volunteers
MA	35.36 ± 1.06	32.53 ± 2.92	33.07 ± 0.80	30.19 ± 1.61
MC	35.01 ± 0.79	30.43 (28.95, 32.79)	33.71 ± 1.34	28.01 (25.68, 29.85)
MP	37.10 ± 0.92	34.30 (31.01, 36.63)	35.70 ± 1.61	28.58 ± 1.32
LA	34.63 ± 1.47	32.16 ± 2.68	33.63 (32.20, 34.33)	29.08 (27.48, 30.49)
LC	35.30 ± 1.28	31.43 ± 2.98	34.47 ± 1.14	27.37 ± 1.35
LP	37.45 ± 0.54	32.23 (31.19, 36.73)	36.62 ± 0.81	29.64 ± 1.32
TLC	35.81 ± 0.42	32.41 ± 1.91	34.42 ± 0.71	28.73 ± 1.02

The table is represented by the average (or median, if the normal distribution is not satisfied) of the T2 value of ROI.

There was a significant interobserver agreement between physicians **, **, and ** (ICC = 0.952). In addition, there was high interobserver reproducibility between observers **, **, and ** (ICC** = 0.983, ICC** = 0.977, ICC** = 0.980).

## Discussion

This article aims to investigate the effects of overuse injuries on ankle talar cartilage in both adults and juveniles. We analyzed the changes in T2 values of ankle cartilage in healthy volunteers compared to athletes, and further expanded our study by recruiting juvenile volunteers and athletes to compare and contrast the characteristics of ankle cartilage T2 values and the distribution of degeneration. Our findings indicate substantial effects of overuse injuries on ankle cartilage, with significant differences in mean T2 values of talar cartilage and its six ROIs across the four groups studied (adult athletes > juvenile athletes > adult volunteers > juvenile volunteers). The difference in the mean T2 value of the talar cartilage between juvenile athletes and juvenile volunteers was higher than the other three groups. These results provide valuable insights into the effects of overuse injuries on ankle cartilage in different populations.

Numerous studies have demonstrated that age and weight have significant effects on cartilage degeneration. Therefore, strict control of inclusion criteria is essential to minimize selection bias. Cartilage T2 value changes in an age-dependent manner, as previously reported in the literature^28^. Following bone maturation, collagen and water content within the bone remain relatively stable, while proteoglycan composition undergoes aging changes^29^. The degradation of proteoglycan aggregates surpasses their synthesis with increasing age, resulting in smaller and fragmented proteoglycan aggregates with a corresponding increase in breakdown products^28,29^. Normally, highly charged proteoglycan aggregates maintain water stability and restrict water movement^30^. However, a reduction in volume and composition of proteoglycan aggregates limits their ability to impede water mobility^29^. The transition zone of articular cartilage contains the highest concentration of proteoglycans, and an asymptomatic increase in T2 values in this zone is associated with aging, which is consistent with previous studies demonstrating an age-dependent increase in water mobility^28^.

Obesity is significantly associated with increased risk of osteoarthritis, particularly in the knee. Some studies have reported that obesity increases osteoarthritis risk by approximately three times^31,32^. The underlying mechanisms linking obesity with osteoarthritis development are multifactorial. Increased joint loading due to weight gain is believed to contribute to the increased risk of osteoarthritis. Additionally, a combination of biomechanical and metabolic factors, such as adipokine-related inflammation-induced cartilage catabolism, are thought to play an equally important role. Individuals with high body mass index (BMI) exhibit higher T1 rho relaxation times in tibiofemoral cartilage compared to those with normal BMI. Pre-exercise tibiofemoral cartilage thickness also decreases with increasing levels of obesity^33^.

Anthony et al. reported elevated cartilage T1 rho and T2 values in runners after a marathon, indicating the occurrence of biochemical changes in articular cartilage. T1 rho values remained high even after three months of reduced activity, while T2 changes were reversible^34^. Meng et al. found that T1 rho and T2 values decreased in all regions of articular cartilage after physiological activities such as running, walking, and stair climbing. The decrease in values was more significant after running than walking, with the exception of femoral cartilage where the decrease was similar for both activities. After stair climbing, T1 rho and T2 values decreased more than after running^35^. In young healthy adults who underwent long-distance running, Cyrus et al. observed significant reductions in mean T2 and T2* relaxation times across all segments of knee articular cartilage^36^. Furthermore, C. Behzadi et al. demonstrated that elite-level professional football players exhibited higher T2* relaxation times in knee articular cartilage compared to age- and BMI-matched amateur athletes^37^.

Previous studies have consistently demonstrated that risk factors such as aging and exercise have an impact on cartilage, aligning with the findings of this study. However, it remains unclear why there is a higher growth rate of cartilage T2 values in the underage group compared to the adult group. We posit that there exist physiological and psychological distinctions between children and adults that could possibly account for the heightened susceptibility to injury observed in children. Factors that make children more susceptible to injury include: children have a greater surface area to mass ratio, children have larger head proportions, children's growing cartilage may be more susceptible to stress, children may not have complex motor skills, children are smaller and vary in size so protective equipment may not be appropriately sized^8^. Resistance training can potentially damage the epiphysis or growth plate in the body of a young weightlifter, leading to early closure^40^. Due to the androgens produced in the body that produce greater strength and speed, which, combined with a more impulsive personality during adolescence, may increase the likelihood of injury^41^.

Recent studies on cartilage imaging modalities have predominantly focused on the knee joint, utilizing T2 mapping and T1 rho imaging. The application of these modalities in sports has been primarily limited to running, both at the professional and amateur levels. Weightlifting athletes, in particular, have shown a negative correlation between increased cartilage thickness in weight-bearing and non-weight-bearing areas of the knee joint, and training onset time. Therefore, children and adolescents experiencing high impact loads may suffer from cartilage damage^10^. To address this gap in the literature, we selected weightlifters as a sample and hypothesized that overuse injuries cause cartilage degeneration, which can be quantified via T2 mapping. Previous studies have reported different findings regarding T2 and T1 rho relaxation times after long-distance running or other physiological activities such as walking and stair climbing. While some studies indicate that these values increase, others report a decrease in all regions of articular cartilage, with subjects in osteoarthritis-related exercise trials exhibiting improved cartilage quality as indicated by reduced mean T2 relaxation times. These contradictory findings in T2 mapping, T1 rho imaging, and T2* mapping may be attributed to the heterogeneity of physical conditions in each individual, such as varying levels of exercise tolerance. The role of potential risk factors, including vitamins, nutritional factors, smoking, bone mineral density, joint surgery, muscle strength, and blood pressure, remain unclear and require further rigorous investigation^38^.

Our study has several limitations. Firstly, the sample size was small. Secondly, the study protocol had limited age stratifications. Thirdly, we only included juveniles aged 15–17 years, but individual growth cycles vary. For instance, females reach peak growth earlier than males. Therefore, adding age groups such as 9–12 years and 12–15 years would enable a comprehensive evaluation of changes in ankle cartilage T2 values with age. Fourthly, while depth-related spatial variations in T2 relaxation time are well-known, we did not consider depth changes when measuring T2 values. We considered the cartilage too thin to be divided vertically^39^. Fifthly, the absence of sample calculations introduces uncertainty regarding the optimal sample size required for accurately detecting meaningful effects or relationships in our study. Finally, chemical displacement artifacts and partial volume averaging of cortical bone or synovial fluid may lead to measurement errors in T2 values due to the thinness of talar cartilage^22^. Future studies should include different types of cartilage, more diverse types of athletes, and consider potential confounding factors. Athletes could be categorized based on their movement patterns and physical demands into three sports cohorts: strength athletes (low dynamic high static sports), high dynamic low static athletes (high dynamic low static sports), and strength endurance athletes (high dynamic high static sports). Groups with similar ages and training years could be selected among these athletes for cross-sectional comparison.

## Conclusion

In conclusion, we strongly recommend the incorporation of functional MRI, specifically T2 mapping, into routine physical examinations of athletes and highly active individuals before symptoms emerge. This would enable maximization of screening for potential pre-injury lesions, allowing for therapeutic measures to be taken to inhibit or delay their development and protect the cartilage health of athletes and highly active individuals, particularly juveniles. This adjustment would enhance the screening process for potential pre-injury lesions, thereby offering reasonable recommendations to athletes and highly active populations, particularly those who are underage.

## Data Availability

The datasets generated and/or analysed during the current study are not publicly available due to participants do not want to disclosure. Data contact: summit-windy@163.com.
